# Trends in childhood intussusception in a Nigerian tertiary hospital

**DOI:** 10.4314/ahs.v24i1.26

**Published:** 2024-03

**Authors:** Uchechukwu Obiora Ezomike, Emmanuel Ifeanyi Nwangwu, Isaac Sunday Chukwu, Sampson Chukwuemeka Aliozor, Chukwuka Arinze Onwuzu, Elochukwu Perpetua Nwankwo, Sebastian Okwuchukwu Ekenze

**Affiliations:** 1 Department of Pediatric Surgery, College of Medicine, University of Nigeria Teaching Hospital, Enugu; 2 Department of Surgery, Alex Ekwueme Federal University Teaching Hospital Abakaliki

**Keywords:** Trends, childhood, intussusception, Nigerian, tertiary hospital

## Abstract

**Background:**

Early presentation, high rate of successful non-operative treatment, low morbidity and mortality in childhood intussusception is common in High and Upper Middle-Income Countries but not in many Lower middle- and Low-income countries.

**Aim:**

To assess the trends in the profile, treatment modalities and outcomes of intussusception in our hospital.

**Materials and methods:**

Retrospective study over a 12-year period divided into two 6-year periods. Data entry/analysis was done using SPSS and various indices were compared between these two periods. Two-tailed t-test for two independent means was used to compare means while two-tailed Fisher exact tests were used to compare categorical variables. Results were presented as tables, means, ranges, percentages and a p-value less than 0.05 was deemed statistically significant.

**Results:**

There was a significant increase in the proportion of successful non-operative treatment (18.6% vs 34%, p=0.03), reduction in the incidence of operative manual reduction (27.1% vs 12.8%; p=0.026), reduction in operative treatment (78.5% vs 63.9%, p=0.034), increased utilization of pre-intervention ultrasound (75% vs96.7%, p<0.0001) and reduction in hospital stay duration (10.47 ±7.95days vs 7.24±4.86 days; p=0.004).

**Conclusions:**

Contribution of successful non-operative treatment to the overall treatment of intussusception significantly increased while that of operative manual reduction significantly reduced and bowel resection showed no change. Preoperative utilization of ultrasonography significantly increased while mean duration of admission reduced significantly, but late presentation, morbidity and mortality rates had no significant changes.

## Introduction

Intussusception is the commonest cause of intestinal obstruction in infancy and early childhood 1,2,3. In many High-Income Countries(HICs) and Upper middle-income countries(Upper MICs) the trend has been towards an early presentation to treatment centers within 24 hours of the onset of symptoms 4,5,6 more successful non-operative treatment 7,8,9 reduced operative treatment 7,9,10 and reduced bowel resection rates 8,9,10. In many Low-Income Countries (LICs) and Lower MICs, however, late presentation, low uptake and low rate of successful non-operative treatment, high rate of operative treatment, and high bowel resection rate are still common 11,12,13,14.

In HICs, it is thought that the constellation of the triad of colicky abdominal pains, vomiting and bloody mucoid substance per rectum is low in incidence 4,6. This classical triad represents a late finding in disease progression 15. It is encouraged that intussusception should be diagnosed before the classic triad manifests 16. In addition, the passage of bloody mucoid substance per rectum is taken as evidence of late presentation 4,5,16,17.

In LICs and lower MICs, the majority of the patients had red currant jelly stool signifying late presentation 3,17,13. Mortality is high in LICs and Lower MICs 12,14,19,20 but low in HICs 7,10,21.

It is thought that over years, with cross-fertilization of ideas and appropriate interventions, the profile and management outcomes of intussusception in LICs and Lower MICs will gradually transit to what is obtainable in upper MICs and HICs.

We, therefore, set out to assess if there are positive trends, or otherwise, in our region toward what is obtainable in upper MICs and HICs over this 12-year study period.

## Materials and methods

This is a retrospective study over 12 years divided into two 6-year periods (January 2010-December 2015 and January 2016-December 2021). The setting is a 500-bed University Teaching Hospital in Enugu State, South-East Nigeria which takes care of pediatric surgical patients within Enugu state and the adjoining states. All children managed for intussusception, as available in the hospital records, were included in the study. Data acquisition involved retrieving various patient indices from patient records. The retrieved indices included age, sex, mean duration of symptoms before presentation, presence of symptoms like abdominal pains, abdominal distension, vomiting, the passage of bloody mucoid substance per rectum, palpable abdominal mass, palpable rectal mass, use of ultrasound for diagnosis, initial hemoglobin level, blood transfusion status, successful treatment modality(non-operative treatment, operative manual reduction or bowel resection), complications, reoperation status, duration of hospital stay, and outcome. Data entry and analysis was done using Statistical Package for Social Sciences (SPSS) version 21(IBM SPSS Statistics for Windows, Version 21.0. Armonk, New York: IBM Corp) and various indices retrieved from the patients' records were compared between these two periods. Two-tailed t-test for two independent means was used to compare means while two-tailed Fisher exact test was used to compare categorical variables. Results were presented as tables, means, ranges, percentages, and a p-value less than 0.05 was deemed statistically significant.

## Results

There were seventy-two cases (forty-seven males) of intussusception over the first six-year period and ninety-four patients (fifty-two males) over the second six-year period. There was a significantt trend towards an increase in the proportion of successful non-operative treatment (18.6% vs 34% p=0.03), reduction in the incidence of operative manual reduction (27.1% vs 12.8%; p=0.026), increased use of pre-intervention ultrasound for diagnosis (75% vs96.7%, p<0.0001) and reduction in the mean duration of hospital stay (10.47 ±7.95days vs 7.24±4.86days; p=0.004) (table 1). There were no significant changes in male-to-female ratio, age at presentation, mean symptom duration before presentation, percentage presenting within the first 24 hours of the onset of symptoms, percentage presenting within the first 48hours of onset of symptoms, frequency of occurrence of the classical triad, bowel resection rate, rate of blood transfusion, mean hemoglobin level at presentation, complication rate, reoperation rate and mortality rate between the two periods. Further details of the results are shown in table 1 below.

**Table 1:** Comparing various indices between the two study periods

	2010-2015	2016-2021	p-value
**Preoperative variables**			
**Total number of cases**	72	94	-
**Age(months)**	Mean age: 8.14±8.28	Mean age: 7.56±7.4	0.36
	Median age: 6	Median age: 6	
	Age range: 3-60	Age range: 3-48	
Male to female ratio	1.8: 1	1.23: 1	0.27
Mean duration of symptoms(days)	3.97±2.93	3.87±2.92	0.84
Percentage presenting within 24hours	19.44% (14/72)	21.3% (20/94)	0.85
Percentage presenting within 48hours	36.11% (26/72)	39.3% (37/94)	1.00
Percentage with abdominal pain	95.8(70/72)	100% (94/94)	0.22
Percentage with abdominal distension	68% (50/72)	51.1% (48/94)	0.49
Percentage with vomiting	100% (72/72)	98.7% (82/83)	1.00
Percentage with passage of bloody mucus	100% (72/72)	98.7% (82/83)	1.00
Percentage with palpable abdominal mass	61% (44/72)	65.4% (55/84)	0.62
Percentage with mass palpable on rectal examination	38.8% (28/72)	32.2% (28/87)	0.41
Percentage with classical triad	58.3% (42/72)	57.8% (55/94)	1.00
Percentage that had ultrasound for diagnosis	75% (54/72)	96.7% (89/94)	**<0.0001**
Mean Hemoglobin level on presentation(g/dl)	9.83±1.71	9.86±1.87	0.88
Perioperative/postoperative variables			
Percentage that was transfused	69.4% (50/72)	70.5% (62/88)	1.00
Percentage successful non-operative treatment	18.5% (13/70)	(34%)32/94	**0.03**
Percentage operative manual reduction	27.1% (19/70)	12.8% (12/94)	**0.026**
Percentage bowel resection	51.4% (36/70)	51.1% (48/94)	0.23
Percentage total operative treatment	78.5% (55/70)	63.9% (60/94)	**0.034**
Percentage with complications	38.3% (23/60)	37.7% (23/61)	0.5
Reoperation rate	7% (5/72)	3.2% (3/94)	0.3
Duration of hospital stay(days)	Range: 1-43	Range: 2-21	**0.004**
	Mean: 10.47±7.95	Mean: 7.24±4.86	
Mortality	7% (5/72)	9.6% (9/94)	0.59

## Discussion

It was observed from the current study that there was no significant reduction in symptom duration at presentation over the 12 years. From other studies, late presentation to the final treatment healthcare facility is still a challenge in many developing countries 18,20,22. The exact reasons for late presentation may not be easily known but are multifactorial and occur at multiple levels 22 and some authors have attributed this to poverty, ignorance, wrong diagnosis, and delayed referrals 17,19.

In HICs, however, the majority of patients present early 4,5,6.

This study also showed an increase in the proportion of patients presenting within 24 hours and 48 hours of the onset of symptoms within the study period but this was, however, not statistically significant. The low proportion of patients presenting within twenty-four hours of the onset of symptoms has also been replicated in other studies in LICs and lower MICs 16,19,23,24 but not in some HICs where the majority present within 24 hours 4,5,6.

There was also no significant change in mean age at presentation across the two periods and this mirrors the findings in some other studies in Africa 11,12,18,25. This, however, is lower than the mean ages recorded in some other countries like Hong Kong where Wong et al 26 had a mean age of 12.5 months, Turkey where Avci et al 8 noted a mean age of 24.83 months, and South Korea where Lee AH et al 7 recorded a median age 18.7 months. The sex ratio remained stable through the study period as there was no significant change in the proportion of males and females affected by intussusception. There remained more males than females and this is similar to findings in some other studies from Africa 12,13,18,20,23 and beyond 7,21,26,27.

The total number of cases managed was more in the second half of the 12 year period but this is not as marked when compared with the doubling of the incidence of intussusception over a 10year period in a study from Ontario Canada 2. In a publication by Gadisa et al 23, there was also a marked rise in incidence over time in the Ethiopian hospital of study. It is difficult to say if tis increase in the current study is due to a real increase in incidence, better health-seeking behavior, better record-keeping, increase in population (from increased birth rate or migration) or a combination of all these factors to variable extents.

There was no significant change in the proportion of patients with the different symptoms of intussusception between the two periods. Also, the proportion of patients with the classical triad of colicky abdominal pains, vomiting and red currant jelly stool remained high throughout the study period. These findings may be explained by the persistent late presentation involving the majority of patients during the 12-year duration of the study. It is encouraged that intussusception should be diagnosed before the classical triad is obvious 15. The rate of classical triad detection was also high in some studies in Africa 3,23 but low in HIs 4,15,28.

The current study saw a significant increase in pre-treatment diagnostic abdominal ultrasound imaging from 75% in the first six years to 96.7% in the second half of the study period. Despite the availability and high utilization of ultrasound, late presentation to the final treatment facility persisted. This may be due to the low index of suspicion in peripheral hospitals where the patients were first seen earlier in the course of the disease. Hence ultrasound is done later in the course of the disease helping diagnosis but rather late to improve outcome. Similar noteworthy increases in the utilization of pretreatment imaging, from 57.5% to 99.3%, were also observed by Kolar et al 9 in Canada in their work published in 2020. Otero et al 21 in USA in their 2019 article also noted a high proportion of pretreatment imaging. Our finding, however, is at variance with some other African studies 10,14,24 where there was low utilization of imaging diagnostic modalities in management of intussusception. From the current study, there seems to be a changing trend toward higher utilization of pretreatment imaging for diagnosis as seen in high-income countries 10. This is an encouraging trend that, however, should be geared towards ensuring the performance of abdominal ultrasound earlier in the course of the disease to assist in earlier diagnosis and subsequent earlier treatment.

The rate of operative treatment in the current study significantly reduced from 78.5% to 63.9% over the two study periods. Some other studies also found a significant reduction in operative treatment in America 27 and Canada 9. Despite the significant reduction in the rate, it is still very high when compared to the rate in some HICs 4,7,21. Our current rate of operative treatment from the second half of the study is however lower than most studies from LICs and lower MICs 1,10,11,13. In some earlier studies 17,19 and even more recent studies 12,20 in Africa, only surgical treatments were offered. Early presentation and introduction of non-operative treatment in LICs will over time likely lead to a reduction in the proportion of operative management to levels seen in HICs. Thanh Xuan et al 16 noted that late hospital admission (≥ 24 hours from illness onset), bloody stool, and presenting with the classic triad of symptoms of intussusception were found as the factors that correlated positively with surgical management outcome. These findings are still very common in our setting.

Bowel resection remained the most common treatment modality over the two periods. There was no significant reduction in bowel resection rate despite the increased success of non-operative treatment. This finding is opposed to some other publications where bowel resection featured less prominently 9,21,26. The high prominence of bowel resection as a treatment modality is likely due to persisting late presentation as also seen in many other African studies 11,14,20,22,23. Bowel resection was also the main treatment modality in some other publications 1,13,14,19,20,29.

The proportion of operative manual reduction as a form of treatment markedly reduced in the second half of this study period when compared to bowel resection. It is thought that as the proportion of successful non-operative treatment increases, the proportion of operative manual reduction reduces. This is because most previously done operative manual reduction would have been treated successfully using non-operative treatment modalities. The number of manual reductions at the surgery also reduced and were less than bowel resection as non-operative treatment increased in some other studies 13,29. The percentage of manual reduction was higher than bowel resection in some earlier studies where there were no non-operative treatment modalities 14,19,23,30. Therefore, when there is no contraindication, non-operative treatment should be encouraged to reduce operative treatment.

The proportion of successful non-operative treatment increased significantly during the study period, though to a lesser degree than noted by another study where Kolar et al in Canada recorded a major increase in the proportion of successful non-operative from 23.4% to 75.2% 9. Generally, the percentage of successful non-operative treatment is still low in many LICs and lower MICs 1,10,24. Earlier presentation for definitive treatments will likely increase this proportion to what is obtainable in upper MICs and HICs 7,10,31.

From the current study, non-operative treatment is increasingly being used but the contribution of successful non-operative treatment to the whole number of successfully treated intussusception is still low. This may still be explained by late presentation leading to a high failure rate while a large number don't qualify for hydrostatic treatment due to peritonitis and massive abdominal distention at presentation. In the study by Otero et al 21 and Ughasoro et al 32, the cost of treatment for those that had surgery was many times more than for those that had no surgery. Hence, in those who qualify, non-operative treatment should be encouraged in lower MICs and LICs. The mean duration of hospital stay in this study was significantly reduced from 10.47 days to 7.24 days despite persisting late presentation in a lot of patients. This may be explained by the reduced hospital stay for the increasing proportion of successful non-operative treatment. This reduction in mean hospital stay is a good development as overall reduced hospital stay translates to less expenditure for the patients at the microeconomic and macroeconomic levels. Otero et al 21 in the USA recorded a mean hospital stay of 2.8 days while Tsolenyanu et al 20 in Togo and Tagbo et al 11 in Nigeria noted 10 days and Chalya et al 12 in Tanzania had 7 days.

There was no significant change in complication rates between the two periods. This may be due to a persistently high rate of bowel resection. Some published studies from other parts of Africa like Gadisa et al 23 in Ethiopia, Chalya et al in Tanzania 12, Bode et al 3 in Nigeria also recorded high complication rates. The reoperation rate did not also change significantly as most of the complications were wound complications that were managed non-operatively. Furthermore, the recurrence rate is low possibly due to persisting high rate of operative treatment as post-treatment recurrence is usually low with operative treatment 26,30 and more with non-operative treatment.

Additionally, the mortality rate is high and did not change significantly. High mortality rates were also noted from other studies 1,10,11,12,14,25. This may be due to persisting late presentation and high rate of operative treatment as some studies 1,23 have associated high mortality with bowel resection while in one of the studies 24 none of the enema-reduced intussusception cases died. Mortality rates, however, are much lower in some other studies especially HICs and upper middle-income countries 7,21,33.

The study is limited because it is a retrospective single-center study. There was also no information on the total number that had non-operative treatment and the percentage that was successful.

We recommend that efforts should be geared towards reducing the duration of symptoms before presentation and maximizing the opportunities for non-operative treatment in Lower MICs and LICs.

Further studies will be geared towards studying the causes of persisting late presentation and late treatment, recurrence rates following various modalities of treatment and success rate of non-operative treatment.

## Conclusions

The contribution of successful non-operative treatment to the overall treatment of childhood intussusception has significantly increased while that of operative manual reduction has significantly reduced and the contribution from bowel resection did not change significantly. The uptake of pre-intervention abdominal ultrasound imaging as a diagnostic method significantly increased. The mean duration of hospital stay was reduced significantly but the late presentation, high morbidity, and mortality rates had no significant changes.
